# Analyzing and Mapping Sweat Metabolomics by High-Resolution NMR Spectroscopy

**DOI:** 10.1371/journal.pone.0028824

**Published:** 2011-12-14

**Authors:** Viktor P. Kutyshenko, Maxim Molchanov, Peter Beskaravayny, Vladimir N. Uversky, Maria A. Timchenko

**Affiliations:** 1 Institute of Theoretical and Experimental Biophysics, Russian Academy of Sciences, Pushchino, Moscow Region, Russia; 2 Institute for Biological Instrumentation, Russian Academy of Sciences, Pushchino, Moscow Region, Russia; 3 Department of Molecular Medicine, College of Medicine, University of South Florida, Tampa, Florida, United States of America; Russian Academy of Sciences, Institute for Biological Instrumentation, Russian Federation

## Abstract

The content of human sweat is studied by high-resolution NMR, and the majority of organic components most often found in sweat of conditionally healthy people are identified. Original and simple tools are designed for sweat sampling from different areas of human body. The minimal surface area needed for sampling is in the range of 50–100 cm^2^. On all the surface parts of the human body examined in this work, the main constituents forming a sweat metabolic profile are lactate, glycerol, pyruvate, and serine. The only exception is the sole of the foot (*planta pedis*), where trace amounts of glycerol are found. An attempt is made to explain the presence of specified metabolites and their possible origin.

## Introduction

Modern high-resolution NMR techniques provide an opportunity to study a wide spectrum of biological objects, including various biological fluids, which are often complex mixtures of organic and inorganic components. The studying of biological fluids by NMR is a promising direction of modern metabolomics (metabonomics), which analyze metabolic profiles to understand the processes occurring in living organisms. Some of the metabolomics aims have applications in diagnostics and pathology identification, such as defining increased or decreased levels of metabolites and detecting the appearance of new components. The analysis of content changes of some components in biological fluids is widely used in medicine. The most studied fluids in clinical practice are blood and urine and, as a rule, their analysis is carried out by routine methods. However, the utilization of high-resolution NMR can be exceptionally important, since ^1^H-NMR spectrum contains the well-resolved signals from almost all physiologically significant components. The entire procedure of fluid analysis by the NMR technique takes approximately 10 minutes (for urine), including the sample preparation, spectrometer tuning, and receiving the spectrum information about organic acids, amino acids, protein components, lipids, sugars, nitrogen containing substances, and others. The relative concentration of components could be easily calculated from the spectrum.

But despite its appealing nature, the use of NMR for the identification of components in biological fluids should not be considered as a substitution for clinical analyses. Instead, high-resolution NMR is an excellent analytical method that provides assistance for the complete and precise diagnosis of disease. However, such an analysis could be a difficult task, since the metabolite profile of a living organism is affected by various factors characterizing the normal functioning, such as the outside temperature (including the seasonal variations), the diet (since consumption of different food can obviously change the metabolic profile), and even the time of food intake [1]. Finally, one should take into account the fact that different pathologies could affect the metabolic profile in a similar way. Needless to say, finding a reliable interrelationship between the peculiarities of a metabolite profile and certain pathology is a difficult task.

In contrast to the vast quantities of literature dedicated to the analysis of blood and urine, the secretions of the sweat gland have been investigated relatively poorly and, therefore, are rarely used in clinical practice. Sweat is a dilute electrolyte solution excreted by the eccrine (sweat) glands in the skin of mammals. Although the primary function of sweating is to control body temperature via evaporative cooling, it has been proposed that some components of male sweat (e.g. androstadienone (4,16-androstadien-3-one)) can also serve as chemosignals that influence the hormonal balance of females, and therefore act as pheromonal stimuli [2].

The lack of wide application of sweat analysis in medicine and biology is explained by the difficulties of sweat sampling in sufficient quantities for analytical work. Although the composition of sweat is primarily water, previous studies have shown that various organic and inorganic compounds are also present. One of the first studies on the sweat composition was presented in a paper by Embden and Tachau published in 1910, where the authors used a microbiological technique to identify the presence of free amino acids, particularly serine, in the sweat samples from patients with febrile diseases [3]. Although these findings on the presence of free amino acids in human sweat were supported and extended by Haugen and Talbert [4], Mosher [5], and McSwinney [6], who measured the concentrations of total amino acids in sweat samples from healthy subjects, and by Hier et al. [7], who determined the concentrations of 10 amino acids in sweat samples from healthy subjects by microbiological assay, the next report on the detailed analysis of chemical content of sweat was published almost 35 years later, where in their 1943 paper, Mickelsen and Key identified the major sweat components, such as chlorine, lactate, urea, creatinine, and uric acid [8]. The systematic investigations of sweat's chemical contents have only started in the last third of the past century [9].

In this work, we report the high-resolution NMR analysis of the composition of sweat samples systematically obtained from different parts of the body using a set of specially constructed tools.

## Results and Discussion

The sweat sampling was carried out using specialized tools described in the Materials and Methods section.

**Figure 1:**
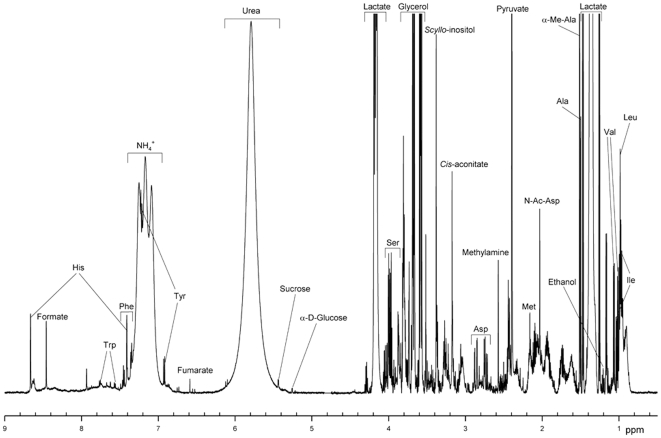
^1^H-NMR spectrum of human sweat from forehead (male).

**Figure 2:**
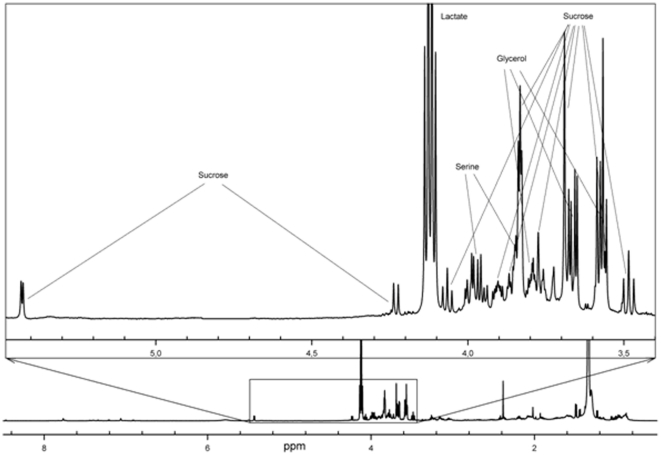
^1^H-NMR spectrum of sweat from human back, «sugar region» is highlighted (male).

**Figure 3:**
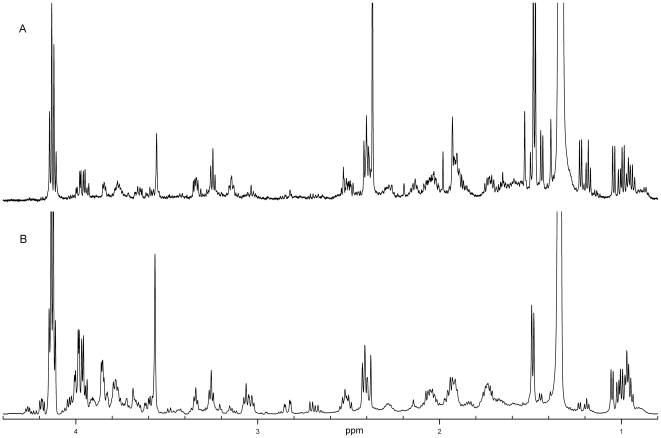
^1^H-NMR spectrum of sweat from child back (A) and male sole of foot (B). In contrast to spectra of sweat from human back and forehead (Figs. 1 and 2), the observed signal of glycerol in the 3.5–3.8 ppm region of NMR spectra was very weak.

Fig. 1 represents the ^1^H NMR spectrum of the sweat sample taken from the forehead, whereas NMR spectra of the sweat samples from adult's back, child's back, and male sole of foot are shown in Figs 2, 3A, and 3B, respectively. As seen from Fig.1, the ^1^H NMR spectrum of the forehead sweat was characterized by intense signals of lactate, pyruvate, and glycerol. Lactate gave the most intense and characteristic signals: the doublet at 1.34 ppm and the quadruplet at 4.12 ppm. One should bear in mind that lactate is present in all human biological fluids, water wash-outs from different animal organs, and in waste products of microorganisms [1], [10]. It is also well-known that the premises inhabited by humans or animals still contain lactic acid, which can be found in the air, on the surfaces, and in the water [11], [12]. Furthermore, one could find negligible quantities of lactate even in sealed vessels containing D_2_O manufactured by various companies running the experiments where the large number of accumulations is needed for the acquiring of the quantitative NMR spectra of the low concentration samples, such as a tear drop etc. [13], [14]. This unique property of lactate could be related to its hygroscopicity and its ability to admix with water at almost any ratios. This determines the abundant accumulation of lactate in water vapor under normal conditions. Due to its omnipresent nature, lactate detection could be used as a simple test for the identification of presence of water-based and protein-based life in different hard-to-reach areas studied by man.

In our work, we refined the assignment the NMR lines reported by Harker *et al*. [9] and also assigned lines to glycerol (one of the most intense lines in the NMR spectrum), serine, lactate and pyruvate, which determine the characteristic profile of the sweat NMR spectrum, but which were not assigned before, although corresponding signals are clearly present in the reported NMR spectrum. It seems that this region of spectrum was not previously analyzed in detail [9], although serine was reported as a major sweat component as early as in 1910 [3].

Glycerol gave a typical spectrum, with multiplets at 3.79, 3.66, and 3.52 ppm. It has a thermal conductivity two times less than water, a boiling point of 290^o^C, and, like lactate, admixes well with water at various ratios. The primary purpose of sweat secretion is to prevent the organism from overheating via fluid evaporation from the body's surface. Glycerol is one of the so-called natural moisturizing factors, which also include various fats, urea, lactic and hyaluronic acids, as well as with other components. These natural moisturizing factors defend the skin surface from drying, thereby maintaining the elasticity and mechanical strength of the epidermis. The presence of glycerol in sweat is due to the lipase activity of skin bacteria hydrolyzing glycerolipids, which are part of the sebaceous and sweat gland secretions. This hydrolysis is the normal physiological process which determines the skin secretion acidity that is crucial for the formation of unfavorable conditions for pathogenic microorganism development [15], [16]. It is also important to note that glycerol is much less abundant in asthenic 3 to 8 year-old children, as followed from our analysis of samples taken from the upper part of the back (Fig. 3A).

**Figure 4:**
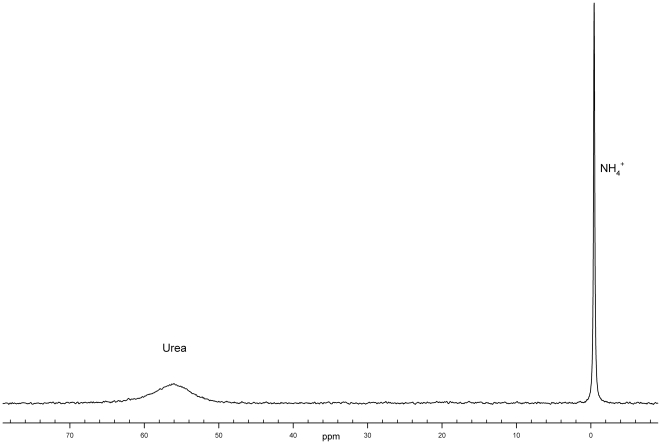
^14^N-NMR spectrum of human sweat from forehead (male).

The next most abundant components in the forehead sweat samples were urea and ammonium cation. Urea gave the wide signal at 5.8 ppm, whereas NH_4_^+^ was characterized by a triplet (J∼50 Hz) at 7.1 ppm. The amount of urea in samples varies in a very wide range (including the complete lack of urea signal in some samples). Similarly, the amount of the ammonium cation is sample dependent. Furthermore, the ammonium cation signal can change its shape and chemical shift in a pH-dependent manner. In the ^14^N NMR spectra of the forehead sweat, along with the broad urea signal at ∼55 ppm there is a narrow ammonium cation signal around 0 ppm (see Fig. 4).

Another distinctive component of sweat was pyruvate, which, in NMR spectra, was characterized by the singlet at 2.37 ppm. The intensity of the pyruvate NMR signal, and, correspondingly, its concentration in the sweat samples varied within the particular limits. There was also a highly sweat-specific signal in the region of the aminoisobutyl acid (α-methyl-alanine) at 1.5 ppm. This signal was observed for all donors, both men and women, in samples collected from for all parts of the body. α-Methyl-alanine is a non-proteinaceous amino acid (the final product of the pyrimidine metabolism), which is known to be excreted with urine by ∼5% of healthy people [17].

There is an almost steady level of free amino acids in the sweat samples (Table 1). Although free amino acids can be found in practically all biological fluids, each biological fluid is typically characterized by an abundance of a specific amino acid or other organic compound(s). For sweat, such a characteristic amino acid is serine, whose multiplet is always detected at 3.96 ppm. Taurine, with its triplets at 3.45 and 3.63 ppm, is a unique characteristic substance for the saliva [18], whereas creatinine and hippuric acid are the unique characteristic signatures of urine [19].

**Table 1:** Chemical shifts of ^1^H NMR and concentrations of metabolites revealed in human sweat.

Chemical shift (ppm) and multiplicity	Metabolite	NMR spectrum	C, mM		
			Forehead^a^	Arms	Forehead^b^
9.68 (t)–2.25 (d)	Acetaldehyde	1D	trace	trace	trace
8.64 (s), 7.39 (s), 4.05 (m), 3.36 (m)	Histidine	1D+2D(COSY)	trace	trace	0.17
8.56 (s), 7.62 (s), 7.28 (d), 6.51 (s)	Urocanat	1D+2D(COSY)	trace	trace	trace
8.44 (s)	Formate	1D	trace	trace	trace
7.76 (d), 7.57 (d), 7.35 (s), 7.31 (t), 7.22 (t), 4.09 (m), 3.52 (m), 3.34 (m)	Tryptophan	1D+2D(COSY)	trace	trace	trace
7.43 (m), 7.38 (m),7.33(m)	Phenylalanine	1D+2D(COSY)	0.26	trace	0.04
7.20 (d), 6.90 (d), 3.94 (m), 3.21 (m), 3.05 (m)	Tyrosine	1D+2D(COSY)	0.27	0.04	0.07
7.14 (t, J = 49.2Hz)	Ammonium cation	1D	21.63*	-	114.85*
6.56 (s)	Fumarate	1D	0.04	0.01	0.01
5.77 (br.)	Urea	1D	30.06*	-	30.77*
5.41 (d)	Sucrose	1D+2D(COSY)	trace	trace	trace
5.23 (d)	*α*-*D-*Glucose	1D+2D(COSY)	0.30	trace	trace
4.66 (d)	*β* -*D-*Glucose	1D+2D(COSY)	0.23	trace	trace
4.35 (m)–2.70 (m), 2.51 (m), 2.01 (s)	*N*- acetyl - aspartate	1D+2D(COSY)	trace	trace	trace
4.26 (m), 3.59 (d), 1.34 (d)	Threonine	1D+2D(COSY)	trace	trace	trace
4.19 (m)–2.51 (m), 2.05 (m)–2.41 (m)	D- pyroglutamate	1D+2D(COSY)	trace	trace	trace
4.15 (q), 1.34 (d)	Lactate	1D+2D(COSY)	234.96	70.87	33.96
4.12 (m), 3.41 (m), 3.33 (m) 2.32 (m), 2.01 (m)	Proline	2D(COSY)	trace	trace	trace
4.06 (m), 3.52 (m), 3.20 (s)	Choline	1D+2D(COSY)	trace	trace	trace
3.97 (m), 3.85 (m)	Serine	1D+2D(COSY)	3.02	0.78	0.58
3.93 (m)–3.20 (m), 3.06 (m)	Cysteine	2D(COSY)	trace	trace	trace
3.92 (m), 2.84 (m), 2.71 (m)	Aspartate	1D+2D(COSY)	trace	trace	trace
3.86 (m), 2.64 (t), 2.20 (m), 2.14 (s), 2.15 (m)	Methionine	1D+2D(COSY)	trace	trace	trace
3.88 (m), 3.54 (m), 3.44 (m), 1.14 (d)	1,2- propanediol	1D+2D(COSY)	trace	trace	trace
3.76 (t), 3.14 (m), 1.88 (m), 1.57 (m)	Citrulline	2D(COSY)	trace	trace	trace
3.82 (m), 2.42 (m), 2.14 (m)	Glutamate	2D(COSY)	trace	trace	trace
3.79 (t), 3.06 (t), 1.95 (m), 1.79 (m)	Ornithine	2D(COSY)	trace	trace	trace
3.79 (q), 1.48 (d)	Alanine	1D+2D(COSY)	0.59	0.18	0.27
3.78 (m), 3.65 (m), 3.56 (m)	Glycerol	1D+2D(COSY)	10.82	0.73	3.02
3.77 (t), 3.25 (t), 1.92 (m), 1.69 (m)	Arginine	2D(COSY)	trace	trace	trace
3.76 (t), 3.02 (t), 1.91 (m), 1.73 (m), 1.48 (m)	Lysine	2D(COSY)	trace	trace	trace
3.74 (m), 1.74 (m), 0.99 (t)	Leucine	1D+2D(COSY)	trace	trace	trace
3.74 (m), 2.41 (m), 2.11 (m)	Glutamine	2D(COSY)	trace	trace	trace
3.76 (d), 1.98 (m), 1.47 (m), 1.26 (m), 1.01(d), 0.94 (t)	Isoleucine	1D+2D(COSY)	0.26	0.04	0.04
3.68 (q)–1.2 (t)	Ethanol	1D+2D(COSY)	Trace	trace	trace
3.61 (d), 2.28 (m), 1.02 (m)	Valine	1D+2D(COSY)	0.43	0.07	0.11
3.56 (s)	Glycine	1D	trace	0.32	trace
3.36 (s)	Scillo-inositol	1D	trace	trace	trace
3.15 (s)	*Cis*-aconitate	1D	trace	trace	trace
2.73 (s)	Dimethylamine	1D	trace	trace	trace
2.56 (s)	Methylamine	1D	trace	trace	trace
2.37 (s)	Pyruvate	1D	6.79	1.79	-
2.23 (s)	Acetoacetate	1D	1.02	trace	trace
1.29 (br.)	Lipid –(CH_2_–)_n_	1D	trace	trace	trace
0.88 (br.)	Lipid and protein (CH_3_–)	1D	trace	trace	trace

*Concentrations of urea and ammonia were determined from ^14^N NMR spectra, trace- trace amount suffuicient for the component identification only.

a,b samples were taken at different times from the forehead of the same 60 years old male donor.

Serine is synthesized from the 3- phosphoglycerate. If necessary, serine could be used as an alternative energy substrate, that, in three steps, is transformed back into 3-phosphoglycerate, which is then included in hydrolysis. This amino acid is a donor of the methyl group, which is transferred onto the tetrahydrofolate during the glycine biosynthesis process. Serine is also an important building block of proteins, phosphatidyl serines, and sphingolipids. Active processes of continuous skin (*integumentum commune*) regeneration demand the synthesis of these substances in large quantities. Serine containing sphingolipids can form up to 50% (by mass) of the membrane lipids, and clearly plays an important role in the skin's protective structures [20]. Apparently, the large amount of serine in skin tissues and sweat gland secretions is a consequence of the aforementioned processes [21], [22], [23].

Free amino acids accumulating in the epidermis are not subjected to reabsorption, and are excreted with the sweat gland secretions due to the exfoliation of dead keratinocytes. Amino acids are the major component of the natural skin moisturizing factors. The specific ionic interactions between keratin and natural moisturizing factors result in the decrease in water mobility and the improvement of the elasticity properties of cornea (*stratum corneum*) due to the reduction of the intermolecular interactions between the keratin fibrils. Furthermore, free amino acids play an important role in the maintenance of the acid-base (pH) balance on the skin's surface [24].

The epidermis of the skin of an organism forms a protective barrier against various unfavorable external coercions of both the physical (mechanical and thermal impacts, ultraviolet irradiation, drying, over-moisturizing, etc.) and the biogenic (development of pathogenic microflora and penetration of its waste products into the organism) nature. The high mechanical strength and elasticity of the stratum corneum are provided mainly by keratin and some other proteins. For example, a special protein filagrin causes the aggregation of keratin filaments. During the keratin synthesis, filagrin is accumulated in a form of the kerato-hyaline granules, and exists in this form until the tightly packed keratin is cross-linked by stable disulfide bonds. Thereafter, filagrin is degraded down to free amino acids in corneocytes. In fact, this protein is the major source of the free amino acids in cornea [25].

Since the sweat and sebaceous glands are parts of the excretory system, they excrete the undesirable products from the organism into the environment, e.g. urea, ammonia, some drugs: griseofulvin, ketoconazole, etc. [26], [27]. For example, some healthy people have rather high levels of sucrose in their sweat (Fig. 2). Usually, sucrose is detected only in the urine of patients with diabetes. Furthermore, the doublet at 1.14 ppm is frequently observed in the NMR spectra of the sweat samples. This doublet most likely belongs to propylene glycol (1,2-propanediol), which is actively used in various medical preparations. Besides the effective accumulation of various excretory products, glomeruli and ducts of the eccrine sweat glands are known to intensely synthesize various proteins, including the large quantities of bactericidal and regulatory peptides. These peptides restrict the development of pathogenic and symbiotic microflora, and determine the normal functionality of the enzymatic and excretory systems of skin cells [28].

The assignments of the vast majority of organic components whose signals are found in the ^1^H NMR spectra of sweat are shown in Table 1. Furthermore, the K^+^, Na^+^, and Cl^-^ concentrations in sweat samples were evaluated from the high-resolution NMR spectra of ^39^Κ, ^23^Na, and ^35^Cl nuclei, respectively. The accuracy of these measurements was at least 10%. The absolute content of these ions clearly depended on the state of the donor body, and varied in a wide concentration range, with the average content being about 60 mM Na^+^, 40 mM K^+^, and 70 mM Cl^-^, which was close to the previously reported data for sweat [5]. Table 1 also represents characteristic concentrations of several organic components found in sweat samples collected from the forehead and arms of the conditionally healthy male donors (40 and 60 years old) and shows that these concentrations can vary in a wide range. Here, sweat samples were taken twice from the forehead of one donor and once from the arm of another donor. Their sweat samples (0.2–0.4 ml) were collected using the specially constructed pipettes. Since in these cases, the volumes of the collected sweat samples were precisely measured, the resulting NMR spectra were quantitatively analyzed providing rather accurate estimates of the concentrations of sweat components. Since the composition and numbers of the eccrine glands at the analyzed body parts (forehead and arms) are similar [26], [27], [29], data shown in Table 1 illustrate a range of normal variation in physiological concentrations of sweat components. This conclusion is further illustrated by the analysis of the sweat content in samples taken from the forehead of the same donor in different day.

Although, the absolute quantitative analysis of the sweat samples collected by the glass rollers is difficult (due to the lack of information on the precise volume of the collected sample), comparison of the signal intensities of different components can be used for the evaluation of their relative concentrations on any selected area of the human body. However, since this method is based on the obtaining of a sample after the moisturizing of any easily accessible body part (e.g., forehead) by distilled water, it is especially useful for the analysis of the sweat content of the patients of any age. Here, the lack of components typically seeing in the norm, the presence of new components, or the obvious deviations in the relative intensities of signals can be used as the biomarker of some pathology. One should remember though that data shown in Table 1 do not represent absolute values and can only be used as basic estimates of the range of concentrations of the component identified by the described methodologies. Furthermore, even for a single conditionally healthy donor, the concentrations of almost all components of the biological liquids are known to vary in a wide range during the day and depend on a number of various factors, including many external conditions [1]. For example, for some blood metabolites, the range of physiologically normal levels can differ by an order of magnitude [29].

The samples from the upper part of chest, the upper part of back, and the arms below an elbow joint had spectra very similar to those described above. The sweat from the lower regions of the back (located closer to sacrum), as well as the axillary and inguinal regions contained larger fractions of fats, and all signals in the proton spectra were broadened in comparison to the sweat spectra from other parts of body. This was probably due to the greater content of triglycerides in those samples determined by the peculiarities of the sebaceous gland distribution on the human body, which is directly related to the distribution of hair bulbs. Typically, a single hair bulb can have the duct openings of 1–3 sebaceous glands. The majority of these glands are located in the skin of the head, the face, the ear canal, the genito-anal region, and the upper part of the back. The minimal density of the sebaceous gland distribution is observed on the backside of hands and feet. Therefore, the low glycerol content found in sweat samples from the feet could be explained by the fact that there are no sebaceous glands on the palm and feet skin [26], [27]. On the other hand, these samples contained large quantities of serine and urocanic acid (see Fig. 3B). The urocanic acid is the histidine metabolism product accumulated in the epidermis due to the failing of the enzymatic system in its catabolism in the keratinocytes [30], [31]. Cells primarily contain *cis*-urocanic acid, whereas the *trans*-form of this acid rapidly isomerizes under ultraviolet irradiation, therefore tempering the unfavorable UV effects. *Cis*-urocanic acid is involved in the UV-induced immunosuppression [30]. Furthermore, this compound plays an important role in the regulation of the acidic homeostasis of the skin's surface. It is also involved in the final differentiation of the epidermal cells. It is excreted with the secretions of endocrine glands and is also exfoliated with the keratin scales [31].

It is important to emphasize here that our analysis of the broadly age-matched groups (i.e., a groups of adults 25–60 years old, and a group of children 3–7 years old) did not reveal any significant gender-related difference of the sweat composition. These findings are in a good agreement with the earlier observations of Harker *et al*., who, based on the analysis of the eccrine sweat samples collected from the larger set of healthy human subjects (30 male and 30 female), came to the same conclusion [9]. On the other hand, the NMR spectra of the sweat samples of children are qualitatively different from the spectra of sweat samples collected from the adult donors, being systematically characterized by the lack of the specific glycerol signals. Since the NMR spectra lacking glycerol signals were collected for children of different age and gander, one can conclude that the absence of glycerol is a characteristic feature of the children's sweat.

In the previous work, sweat samples were collected by the undefined instruments in a room with a fixed temperature [9]. In our study, the developed tools provided a possibility to collect sweat samples at any time, any place, any body part, and at any emotional state of the donors. In fact, the entire procedure of sample collection usually took less than 5 minutes, and at several occasions, samples were collected directly at the donor's working place (based on the preliminary agreement). Quantitative analysis and assignment of the 2D-NMR lines were performed using the more concentrated sweat samples collected by the described pipette during the extremely hot summer of 2010. NMR spectrum shown in Figure 2 is an illustrative example of the normal sweat spectrum. Despite the variations in the intensities of some signals seen in the samples collected from different body parts of different donors, we hypothesized that the sweat composition of the age-matched donors is gender-independent and is qualitatively invariant when samples are collected from the forehead, back, arms and chest. By qualitatively invariant we mean that there is no appearance of new components and complete disappearance of existing components in the NMR spectra. These conclusions are generally supported by the data reported by Harker *et al*. [9] and are independent of the number of donors analyzed. In the norm, there is a rather broad variation in the relative content of various components of different biological fluids. For example, for some blood metabolites, the range of physiologically normal levels can differ by an order of magnitude [32].This variability is a well-known physiological reality, which is determined by the numerous factors, such as peculiarities of diet, emotional state, and mode of the day. In the case of sweat, the emotional background (pain, fear, or any uncomfortable conditions) is known to affect at least the perspiration intensity and therefore the amount of sweat.

Therefore, the fact that Harker *et al*. [9] did found variations in some components is rather expected since for several biochemical quantifications of biological fluids used by the modern medicine to characterize the norm, the allowed difference between minimal and maximal values may vary by at least an order of magnitude.

On the other hand, NMR spectra of different body fluids are characterized by some characteristic features and the overall spectral profile allows the experienced NMR spectroscopist to differentiate between spectra of tear, urine and saliva. These differences are based not on the variation of minor components, but on the variations between the contents of the major components, which, in fact, determine the characteristic spectral profile. Although intensities of signals related to minor and even some major components may slightly vary from spectrum to spectrum, the overall characteristic shape of the spectrum is typically preserved. However, the overall spectral shape could drastically change if the relative intensities of major components would be strongly perturbed. This is exactly what we observed for the spectrum taken for the sweat sample from the sole of the foot (*planta pedis*), where only trace amounts of glycerol were found. In all other cases, sweat spectra where qualitatively similar.

In summary, the high-resolution ^1^H NMR revealed that the sweat samples obtained from different parts of body of conditionally healthy donors generally possessed a qualitative similarity. However, there was a noticeable variation in the individual signal intensities, which is rather typical for all biological fluids [1]. This suggests that the sweat metabolic profiles are relatively conserved in various parts of the human body. The only exception is the feet regions, which are characterized by a unique metabolic pattern. These observations can simplify the procedure of sweat sampling for patients with certain pathologies, since changes in the metabolic profiles can be detected in samples collected from the “patient-friendly” regions, e.g., from the forehead or the upper parts of the chest or the back. However, we believe that for the analysis of pathological changes in the sweat content during the course of illness, sweat samples should be collected from the same body region. Furthermore, we demonstrated that our tools can be used for the fast (3–5 min) collection of sweat samples in the patient-friendly environments and from the patient-friendly body regions, even from the patients with very serious conditions. This is an important advantage, since in the majority of studies reported so far, the sweat samples were collected in the specially designed hot rooms (e.g., at the conditions of 43.3°C and 65% relative humidity [9]). Obviously, such conditions are not safe for many patients.

Due to the presence of a broad set of different low molecular weight organic compounds, and due to the development of new instruments allowing sample collection even from the patients with very serious conditions, the sweat could be considered as a good candidate for the successful diagnostic of some pathology, along with urine and blood.

## Materials and Methods

The ethics approval for this study was obtained from the Ethics Committee of the Institute of Theoretical and Experimental Biophysics, Russian Academy of Sciences (Pushchino, Moscow region), where all the participants were recruited and human experimentation was conducted. Informed written consent was obtained from all the participants involved in this study. Some of the participants were minors and therefore have had a reduced capacity/ability to consent. In these cases, the informed written consent was obtained from the close relatives (parents) on the behalf of participants whose capacity to consent was reduced.

**Figure 5:**
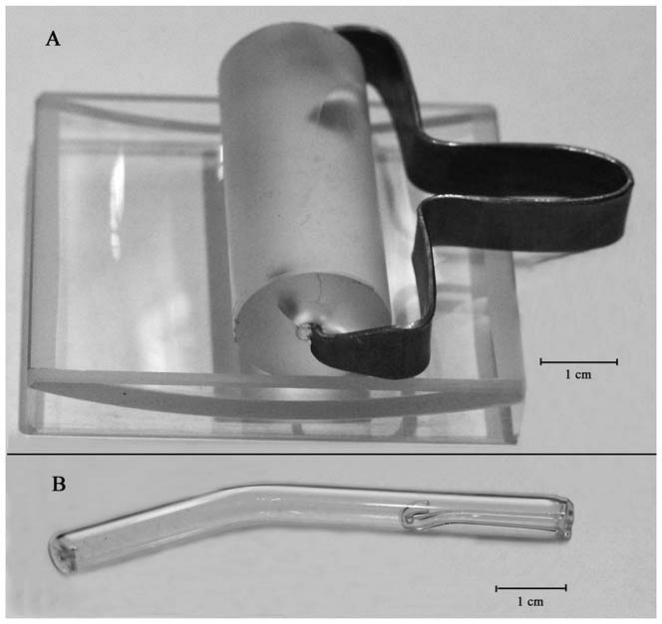
Special tools for sweat collection - glass roller and the holder (A) and special pipette with reverse capillary (B).

Sweat samples were collected by tools designed in our laboratory. These tools include glass rollers with dull surfaces and holders for sample collection when the sweat quantity is limited (Fig. 5A), and special pipettes with reverse capillaries for sweat drop collection in the case of the profuse sweat secretion (Fig. 5B). In order to minimize the amount of impurities in the test specimens, glass instruments were utilized. Before implementation, they were washed with acid and alkali, thoroughly rinsed with an excess of distilled water, and finally heated to 200^o^C–220^o^C to burn the remaining organic components.

To collect a sweat sample from a body region, a dull glass roller (with a diameter of 15–20 mm and a length of 50 mm) mounted on a special holder is rolled along the desired body region (about 100 cm^2^) and then washed several times within a special concave tray with ∼0.65 ml of D_2_O (99.9%, Cambridge Isotope Laboratories, Inc.). The complete sample was then accumulated in the middle of this concave tray, which simplifies the sample collection by the automatic pipette, and the collected sample volume was sufficient for the analysis. In the case of the low sweat secretion, the analyzed body region was moisturized prior to the sample collection using a sterile spray gun filled with the known amount of distilled water. To eliminate excessive contamination of the samples with epidermal cells, collected samples were centrifuged (centrifuge CM50, 10 min, 16000 rpm) and then were transferred to a standard NMR tube. The minimal sample volume used for NMR analysis was 0.56 ml. In the case of the profuse sweat secretion, the mentioned above special pipette was used (Fig. 5B). The reverse capillary of this pipette allowed us to collect up to 0.6 ml of sweat per single use from the surface of the analyzed body region (forehead, chest, back, etc.) due to the wide contact area (∼1 sm^2^) and the entrance diameter of ∼1 mm. Since in this case the collected sweat volume is known, concentrations of the sweat components can be estimated from the signals seen in the NMR spectrum. The errors in the evaluation of relative integral intensities of NMR signals did not typically exceed 10%. This is because of the fact that the low intensity signals were compared to the high signals of the lactate methyl group (a doublet of ^13^C-satellites). Since these lactate methyl group signals typically constituted 1% of the major signal intensity, they were reliably measured. Note that irrespectively of the method used for the sweat sample collection the donor was not additionally treated to promote the artificial sweat secretion. This is important for the unperturbed and unbiased study of patients.

Both one-dimensional (1D) and two-dimensional (2D)-NMR spectra were acquired on a Bruker Avance III 600 spectrometer operating at a frequency of 599.93 MHz (^1^H) and a probe temperature of 298 K. The samples were placed in NMR tubes with diameters of 5 mm. The pulse sequences used in the experiments were standard pulse sequences from the Bruker NMR pulse sequence library. The 1D-pulse sequence WATERGATE (WATER suppression by GrAdient Tailored Excitation) was applied for the suppression of proton signals from water. This method is based on the single symmetrical spin-echo technique, combined with the two symmetrical pulse gradients of the magnetic field. The free induction decay (FID) was collected into 64K data points using an acquisition time of 1.95 s. The spectrum width was 9000 Hz and the 90° pulse width was 11 microseconds. Furthermore, the 2D COSY sequence COSYGPPRQF was used for the refinement of peak assignments. The number of acquisitions was chosen 100–1000 (for the 1D-spectra) and 4–16 (for the 2D-spectra) depending on the sample concentration to obtain sufficient signal-to-noise ratio. The AMIX program database (Bruker) and the internet database HMDB (Human Metabolome Database) were used for peak assignments of different organic components.

For acquiring the ^39^Κ, ^23^Na, and ^35^Cl NMR spectra, the simplest pulse sequence ZG was used. The FID was acquired with 0.4 s. In the case of the nucleus with the lowest sensitivity, ^39^K (27.99 MHz) 1024 transients were acquired using a spectral width of 1600 Hz and a 90°-pulse width of 63 µs. For ^23^Na (158.67 MHz) data were acquired with 128 transients, a spectral width of 3200 Hz and a 90°-pulse width of 28 µs. Finally for ^35^Cl (59.97 MHz) data were acquired with 128 transients, a spectral width of 3500 Hz and a 90°-pulse width of 38 µs. The concentrations of K^+^, Na^+^, and Cl^-^ ions were devised from the NMR spectra of ^39^Κ, ^23^Na, and ^35^Cl nuclei by measuring the integral intensities of signals in the sample spectra and in the standard solution containing 20 mM NaCl and 20 mM KCl. Furthermore, the influence of the aforementioned components on the signal intensity was investigated. It was shown that signal intensity values of the components were proportional to their concentrations in a range of 20-200 mM.

The quantitative analysis of the nitrogen-containing components was performed using 14N (43.34 MHz) NMR. Data was acquired with 1024 transients, a spectral width of 4500 Hz and a 90°-pulse width of 48 µs. Each transient had an acquisition time of 0.2 s. The FID was registered with acquisition time of 0.2 s. The concentrations of urea and ammonia were determined from the ^14^N spectra of the standard solution containing 100 mM urea.

For all these nuclei, FIDs were collected into 2K data points which were zero filled by factor of 2 and the exponential broadening of 5 Hz was applied prior to the Fourier transformation.

Sweat was collected from 10 conditionally healthy donors, three children (3, 5, and 7 years old), two women (25 and 45 years old) and five men from 25 to 60 years old. All donors are citizens of Pushchino (Moscow region, Russia). All of donors were subjected to the clinical and biochemical blood analyses, which did not reveal noticeable deviations from the norm.

The majority of experiments were repeated several times. Most attention was paid to the easily accessible body areas (forehead, the upper part of chest, the upper part of back, the lower part of back, hands in the biceps region). Each experiment was performed under the conditions defining the normal (for each donor) sweat secretion. A special request was placed to exclude the use of the deodorants and cosmetics at least one day prior to the sweat sample collection from the forehead and armpits. The process of the sweat collection from each donor typically took ∼3 min, and 7–10 min was required to prepare the sample. Therefore, the NMR analysis was performed in 10–15 min after the sweat sample collection from a donor. The qualitative analysis was performed following the sweat sample collection from the members of the laboratory in the working environment. Samples were collected using the described above pipettes during the abnormally hot summer of 2010. Sample volumes were typically in a range of 0.2–0.4 ml and the collection time did not exceed 5 min. The concentrations of organic components were determined using the calibration spectra of citrate with known concentration (2.4 mM) measured at the same conditions.
